# Transcriptional slippage in bacteria: distribution in sequenced genomes and utilization in IS element gene expression

**DOI:** 10.1186/gb-2005-6-3-r25

**Published:** 2005-02-15

**Authors:** Pavel V Baranov, Andrew W Hammer, Jiadong Zhou, Raymond F Gesteland, John F Atkins

**Affiliations:** 1 Department of Human Genetics, University of Utah, Salt Lake City, UT 84112-5330, USA; 2 Bioscience Institute, University College Cork, Cork, Ireland; 3 Current address: Gene Technology Division, Nitto Denko Technical Corporation, 401 Jones Road, Oceanside, CA 92054, USA

## Abstract

**Background:**

Transcription slippage occurs on certain patterns of repeat mononucleotides, resulting in synthesis of a heterogeneous population of mRNAs. Individual mRNA molecules within this population differ in the number of nucleotides they contain that are not specified by the template. When transcriptional slippage occurs in a coding sequence, translation of the resulting mRNAs yields more than one protein product. Except where the products of the resulting mRNAs have distinct functions, transcription slippage occurring in a coding region is expected to be disadvantageous. This probably leads to selection against most slippage-prone sequences in coding regions.

**Results:**

To find a length at which such selection is evident, we analyzed the distribution of repetitive runs of A and T of different lengths in 108 bacterial genomes. This length varies significantly among different bacteria, but in a large proportion of available genomes corresponds to nine nucleotides. Comparative sequence analysis of these genomes was used to identify occurrences of 9A and 9T transcriptional slippage-prone sequences used for gene expression.

**Conclusions:**

IS element genes are the largest group found to exploit this phenomenon. A number of genes with disrupted open reading frames (ORFs) have slippage-prone sequences at which transcriptional slippage would result in uninterrupted ORF restoration at the mRNA level. The ability of such genes to encode functional full-length protein products brings into question their annotation as pseudogenes and in these cases is pertinent to the significance of the term 'authentic frameshift' frequently assigned to such genes.

## Abstract 2

To find a length of slippage-prone sequences at which selection against transcriptional slippage is evident, the transcription of repetitive runs of A and T of different lengths in 108 bacterial genomes was analyzed. IS element genes were found to exploit transcriptional slippage for regulation of gene expression.

## Background

During transcription, RNA polymerase catalyzes incorporation of nucleotides into growing RNA chains on the basis of complementarity to the DNA template. While transcribing long poly(A) or poly(T) tracts, however, slippage or 'stuttering' (also known as pseudo-templated transcription) occurs, with the resulting incorporation of one or more extra nucleotides or occasional lack of a base or two corresponding to the run of repeat bases. Transcription slippage was first reported from *in vitro *studies [1], and investigated *in vivo *later [2]. Although sequences that are able to cause efficient transcriptional slippage occur infrequently in genomic DNA, they have been found and a functional role has been assigned to some of them. For example, transcription slippage is utilized for regulation of the *Escherichia coli pyrBI *and *codBA *operons and occurs shortly after transcription initiation when special conditions apply [3,4].

When a transcription slippage-prone ('slippery') sequence occurs in a coding sequence, the mRNA products are heterogeneous. In such an mRNA population, the sequence downstream of a slippery pattern generally occurs in all three different reading phases relative to the reading frame 5' of the slippage-prone sequence. Translation of these mRNAs yields protein products that differ in their amino-acid sequence downstream of the slippery sequence. For genes encoding a single functional protein product, the presence of slippery sequences is expected to be detrimental, as it is likely to squander cellular resources to synthesize unwanted, or in some instances even deleterious, products. Aberrant forms of beta-amyloid precursor protein and ubiquitin B found in Alzheimer's and Down syndrome patients are associated with molecular misreading, whose mechanism is likely to be transcriptional slippage [5]. Moreover, this type of molecular misreading was suggested to be relevant to the aging process [6-8]. Transcriptional slippage in the human *APC *gene (in addition to replicational slippage [9]) has also been proposed as a cause of colorectal cancer [10].

There are, however, at least two situations where transcriptional slippage inside a coding region can be advantageous. One is where a frameshift mutation occurs in the coding sequence and transcription slippage at a nearby site permits synthesis of a proportion of mRNAs in which a non-templated nucleotide(s) compensates for this mutation, thereby restoring the original framing. An example involving a single nucleotide deletion occurs in *apoB*, the human gene in which defects cause familial hypobetalipoproteinemia. In addition to encoding the expected truncated dysfunctional product, about a tenth of the product is full length as a result of its mRNA template having an extra A inserted in a run of eight As [11,12]. A similar situation was recently reported for the canine *AP3B1 *gene [13].

A second situation in which transcription slippage has a positive outcome is when it leads to synthesis of more than one useful product from a single gene - during expression of the P gene in paramyxoviruses, for example. The best-studied example is in Sendai virus, where a specific number of untemplated Gs are inserted at the position corresponding to the slippery site (reviewed in [14]). Remarkably, this process depends on a hexanucleotide phasing of the slippery sequence relative to the end of genome and this is modulated by viral protein N [15]. In addition to its involvement in paramyxovirus decoding, transcriptional slippage is used for the synthesis of additional functional proteins in other viruses, such as Ebola virus [16-18].

**Figure 1:**
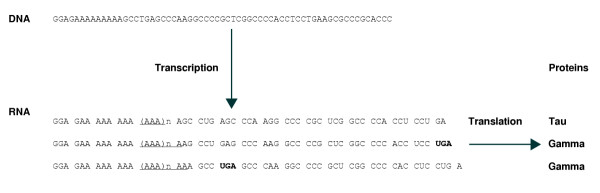
A scheme for the nonlinear expression of *Thermus thermophilus dnaX *via transcriptional slippage. Transcription of *dnaX *results in synthesis of a population of mRNAs in which the sequence 3' of the slippery AAAAAAAAA is framed in different molecules in all three reading frames relative to sequence 5' of the slippery motif.

Utilization of transcriptional slippage is not limited to viral genes. Highly efficient transcription slippage in the decoding of the cellular *dnaX *gene of *Thermus thermophilus *results in 50% of the product being shorter than the 'standard' product [19]. This gene has a run of nine As in its sense strand seven-eighths of the way through its coding sequence. During transcription, RNA polymerase synthesizes mRNA that contains poly(A) runs of variable length. When the number of As is equal to the templated 9 or 9 + 3n, the full-length product, the DNA polymerase III tau subunit, is synthesized. When the number of As is anything else, for example 8, 10, 11, 13, the translating ribosomes encounter a 3' stop codon located close to the poly(A) run. They terminate, resulting in the synthesis of a shorter product (Figure 1), the gamma subunit of DNA polymerase III, which has distinctive functional properties [20,21]. In some other bacteria such as *E. coli *[22-24] and its close relatives [25,26], *dnaX *also encodes both subunits, but the shorter one is synthesized via ribosomal frameshifting instead of transcriptional slippage. The same end result can be achieved by nonstandard events at different levels of readout [19].

Another example of the use of transcriptional slippage was recently reported in the decoding of the *Shigella flexneri mxiE *gene which encodes a transcription activator [27]. *mxiE *consists of two overlapping open reading frames (ORFs), *mxiEa *and *mxiEb*. Transcriptional insertion of an additional non-templated nucleotide at the run of Us results in a proportion of the mRNAs having *mxiEa *and *mxiEb *in the same reading frame [27]. Therefore, in contrast to *T. thermophilus dnaX *transcriptional slippage, where the novel product is shorter than the product of standard decoding, *mxiE *transcriptional slippage is required for synthesis of the longer protein product.

Transcription slippage-prone sequences are expected to be under-represented in coding regions [2], because functional utilization of such sequences is unlikely to be common. The recent dramatic increase in the number of sequenced bacterial genomes provides an opportunity to perform wide-scale analysis of whole kingdoms of life [28]. The current study explores whether long runs of As or Ts are indeed avoided in the coding regions of 108 sequenced bacterial genomes, and where such runs do occur, whether they play a positive functional role in gene expression.

## Results

### Distribution of homopolymeric A and T runs in bacterial genomes

If any sequence pattern is randomly distributed in a genomic sequence, the following equation should be satisfied:

Pc/Pg  Nc/Ng

**Figure 2:**
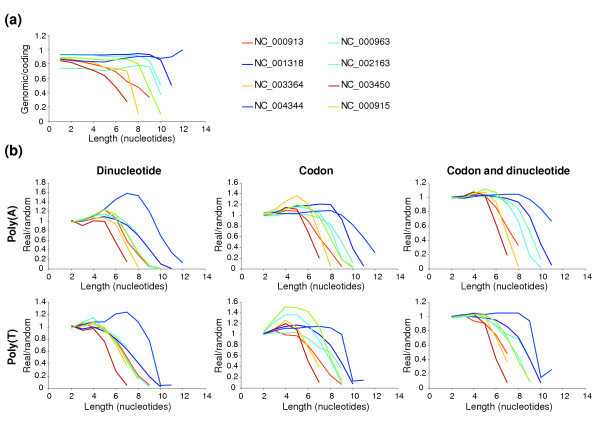
Analysis of the distribution of runs of As and Ts in selected genomes. Run length is indicated on the *x*-axis; the ratio of pattern occurrence on the *y*-axis. **(a) **Ratio of occurrences in coding regions and in entire genomic sequences. **(b) **Ratio of occurrences of A runs in real genomes and average occurrence in 1,000 randomized genomes. Biases preserved during the randomization procedure are indicated above each pair of graphs. Accession numbers are as follows: NC_000913 *E. coli *K12; NC_000915 *Helicobacter pylori*; NC_000963 *Rickettsia prowazekii*; NC_001318 *Borrelia burgdorferi*; NC_002163 *Campylobacter jejuni*; NC_003450 *Corynebacterium glutamicum*; NC_003364 *Pseudomonas aeruginosa*; NC_004344 *Wigglesworthia glossinidia*.

Where Pc is the number of pattern copies in coding regions, Pg is the number of copies in the whole genome, Nc the number of nucleotides in coding regions and Ng the size of the whole genome. We have analyzed the ratio Pc/Pg for 118 published eubacterial and archaeal genomes for homopolymeric A or T patterns of different lengths (see Additional data file 1). An example of such an analysis for a few representative genomes is illustrated in Figure 2a. For several genomes, a sharp reduction in Pc/Pg is evident during transition from the patterns containing *n *number of As or Ts to the patterns containing *n *+ 1 As or Ts. The position of the transition is different among the genomes analyzed. A sharp transition is evident only for AT-rich bacterial genomes; in GC-rich bacterial genomes the existence of long A/T runs has a low probability (if random) [29]. Therefore, they are more likely to occur if there is positive selection. In some AT-rich genomes, however, there is no transition in the Pc/Pg ratio at any length (for example, *Borrelia burgdorferi*). This suggests that such organisms have developed a mechanism to suppress transcriptional slippage at long runs of As or Ts. Indeed the frequency of 9 A/T or 10 A/T runs in such genomes is about one per gene.

Comparison of poly(A) and poly(T) occurrence in genomic sequences versus coding regions has two disadvantages. First, runs of As cannot be discriminated from runs of Ts at the level of genomic sequences. Second, such runs could have a positive or negative role(s) outside of coding regions. For example, long runs of Ts can serve as parts of transcriptional terminators, although poly(T) runs do not have to be uninterrupted for this purpose [30]. In addition, the occurrence of A and T runs can be affected by dinucleotide bias, codon usage and amino-acid composition of encoding proteins.

To minimize the influence of these factors on our analysis, we used another approach to estimate the distribution of such patterns. A thousand random genomes were generated for every genome shown in Figure 2a using the following rules: protein sequences from the real genomes were preserved, but the codons encoding the amino acids were randomized, taking into account codon usage. Such random genomes are relieved of selective pressure to avoid slippery sequences. A similar approach was previously used for statistical analysis of frameshift-inducing patterns in *E. coli *[31] and secondary RNA structures in bacterial genomes [32]. In addition, we used randomization approaches that preserved dinucleotide bias and both dinucleotide bias and codon usage using the DiShuffle and CodonDishuffle programs developed by Katz and Burge [32]. Figure 2b shows the distribution of A/T runs in such random genomes compared to the real genomes. If there were no selective pressure on a particular pattern, its occurrence in random genomes would be similar to its occurrence in a corresponding real genome. If there were negative selection against a particular pattern, it would occur more frequently in random genomes than in real ones. This analysis confirmed our general conclusion that runs of As and Ts of a certain length are avoided in some prokaryotic genomes, but the length of the pattern that is likely to be harmful varies among different genomes. Consequently, such patterns are significantly under-represented in AT-rich genomes.

Interestingly, in the genome of *Wigglesworthia glossinidia*, A/T patterns of any length occur with the same frequency in coding and noncoding regions, suggesting that transcriptional slippage is not possible in this species at patterns of any length. However, when the occurrence of such patterns is compared with their occurrence in random genomes, a negative selection is evident for patterns of exceptional length. This suggests that very long patterns have a negative effect not associated with transcriptional slippage.

### Functional roles of transcriptional slippage

**Figure 3:**
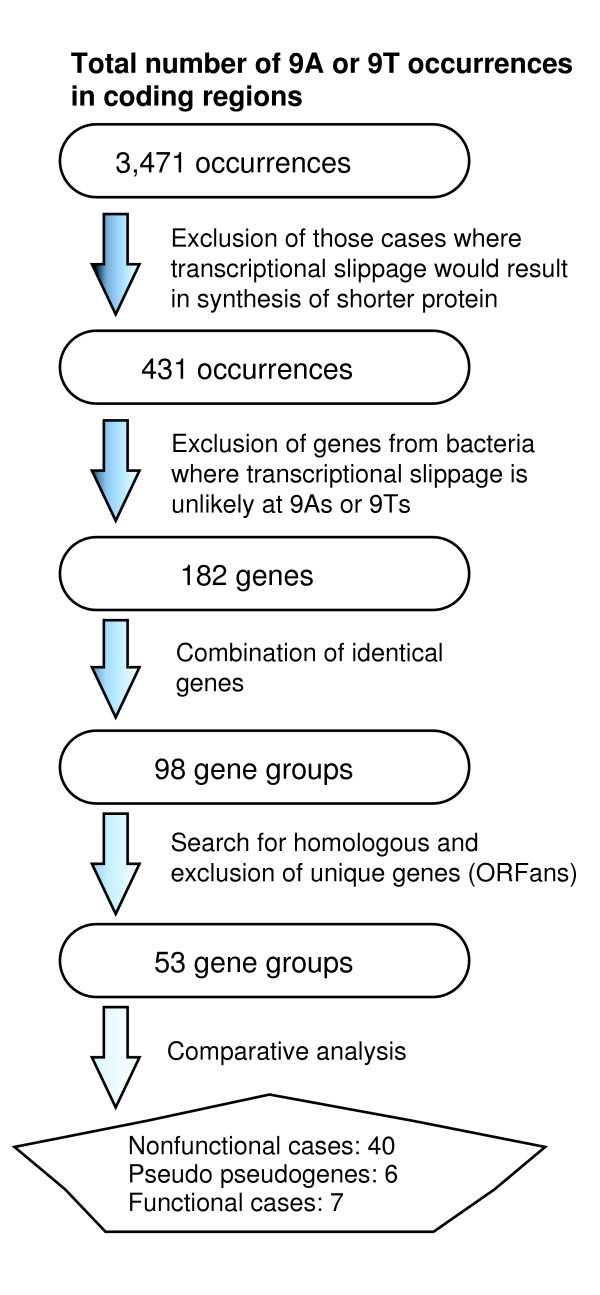
Scheme for functional analysis of slippery patterns in coding sequences.

The next step was to find occurrences of transcriptional slippage and to investigate, using comparative sequence analysis, whether they are likely to have any functional role. The scheme of this analysis is shown in Figure 3. We searched for occurrences of 9As and 9Ts in protein encoding genes. Only those genes were selected where transcriptional slippage would result in synthesis of a protein which is larger than the counterpart generated by standard decoding. When transcriptional slippage results in the synthesis of a truncated product, as in decoding *T. thermophilus dnaX*, it is difficult to predict functional importance on the basis of comparative sequence analysis, as there is no extensive 'new' coding sequence suitable for such an analysis. The next filter was the exclusion of genes from bacteria where transcriptional slippage is unlikely to occur on runs of 9As and Ts. Organisms with AT-rich genomes that do not demonstrate selection against 9A and 9T sequences within their coding regions may have evolved to suppress transcriptional slippage on 9A and 9T and are unlikely to exhibit it. To select bacteria in which transcriptional slippage on 9A and 9T is unlikely, we first determined the number of genes containing 9A and 9T. For those bacteria where this number was higher than the threshold number 20 (we assumed that it is unlikely that transcriptional slippage can be utilized by more than 20 genes in the same species) we searched for evidence of negative selection against these sequences. If such sequences were not under-represented, corresponding bacteria were considered as those where transcriptional slippage is unlikely to occur on 9A or 9T runs. Genes from such bacteria were excluded from further analysis.

The remaining pool of genes contained some identical genes. Some of these exist in multiple copies inside the same genome whereas others are identical because they derived from genomes of highly related species. Such identical genes were combined to reduce redundancy. In the list of these genes (2) only one representative is given for each group of identical genes. The products of those genes that can be generated by transcriptional slippage were compared to each other using tBLASTn [33], and to those derived from other sequences present in sequenced bacterial genomes. Genes that produced no significant sequence similarity were considered as ORFans [34,35]. Since ORFans are not suitable for comparative analysis, they were excluded from further analysis (shown in gray in 2). The number of gene groups for which homologs were found is 53.

**Figure 4:**
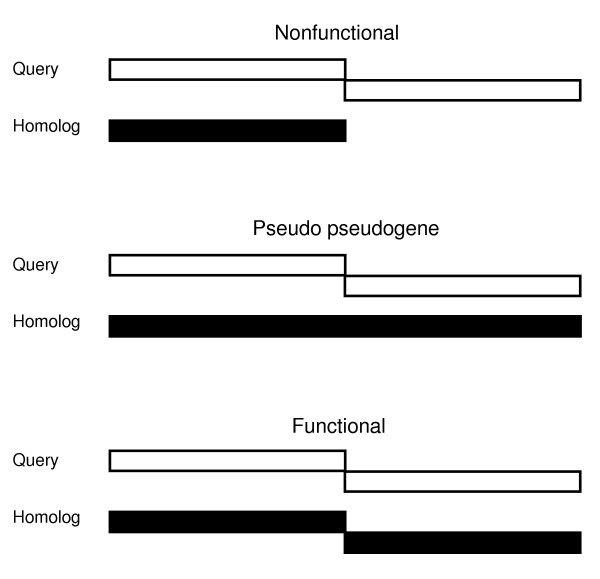
Different types of ORF organization for genes sharing sequence similarities.

The likelihood of functional utilization of transcriptional slippage was estimated using comparative sequence analysis. According to the scheme utilized (Figure 4), we consider transcriptional slippage patterns likely to be functional if the organization of ORFs fused by transcriptional slippage is the same in at least two non-identical sequences sharing significant sequence similarity. We have not found evidence of functional utilization of transcriptional slippage for 40 cases (shown in blue in 2). Most probably, although transcriptional slippage is likely to occur during expression of these genes, it has no significant detrimental effect. This result is consistent with our previous finding that sequences that direct significant levels of frameshifting in the *E. coli *genome may occur without apparent function [31]. Six cases were found where protein products expressed by transcriptional slippage have homologs encoded in a single ORF in genes from other species.

**Figure 5:**
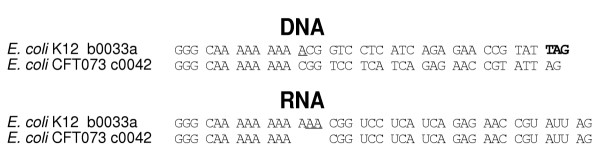
Codon alignments of DNA and mRNA sequences of orthologous genes from two different strains of *E. coli*. In the DNA, an A causing a frameshift mutation is underlined. In the mRNA, a tandem A inserted by transcriptional slippage which results in ORF restoration is underlined.

One example is shown in Figure 5. Such genes are normally considered as pseudogenes, because their ORF is disrupted. However, transcriptional slippage should result in the synthesis of normal functional protein and consequently such genes should not be treated as inactive as a result of frameshift mutation. These genes are shown in green in 2. In seven cases (red in 2) homologs were found with both a conserved organization of the overlapping ORFs and a conserved pattern of 9As in the overlapping regions. Among them, six cases derive from IS elements whose total number of copies is 27. One group is composed of the *mapW *genes from *Staphylococcus aureus *strains; *mapW *is a functional candidate derived from a non-mobile element.

Transcriptional slippage was recently found in the *S. flexneri *pathogenicity-encoding plasmid that carries the *mxiE *gene [27]; it is not included in the 108 sequences of complete genomes downloaded for the present study (even though the chromosomal sequence was included).

## Discussion

We have obtained an initial view of the distribution and functional utilization of simple transcriptional slippage sites in bacterial genomes performed on a multiple-genome scale. The data obtained demonstrate that runs of As and Ts, which result in efficient transcriptional slippage, are significantly underrepresented in coding regions of AT-rich genomes. One likely reason for this underrepresentation is the 'slippery' nature of such sites. In addition to transcriptional slippage, these sequences are likely to be hypermutable as a result of slippage during replication. This also contributes to negative selection against these sequences. It has previously been shown that in eukaryotes short repetitive sequences of specific length are usually under-represented in coding regions compared to noncoding regions [36]. The implication is that such sequences are susceptible to frameshift errors at the DNA level. We cannot distinguish whether the reason for negative selection against A or T runs is slippage at the replication or transcriptional level or at both. Our approach to finding genes where transcriptional slippage is functionally utilized can, however, discriminate it from replicational slippage in some instances. Since we deal with those cases where sequence extension after a slippery pattern in a shifted reading frame is conserved among several homologs, it is very likely that this extension is expressed. Theoretically, its expression can be achieved as a result of replicational and/or transcriptional slippage. In the first case, the result would be the existence of a population of bacteria with heterogeneous genomes where different members of such a population would have a different number of nucleotides within a repetitive run, as previously described for several occurrences in the *Campylobacter jejuni *[37]. We have found several such examples for the group of genes that we classified as 'pseudo pseudogenes' (an example is in Figure 5).

**Figure 6:**
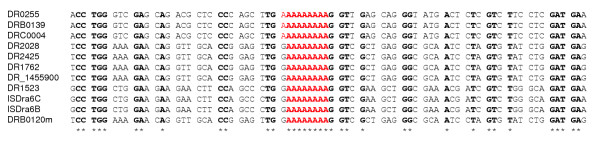
Alignment of a portion of *Deinococcus radiodurans *IS elements containing a run of nine or eight As. Universally conserved residues are in bold, runs of As are in red. The alignment was built using Clustal [54].

If a specific run of 9As or 9Ts occurs within a number of homologs and the length of such run is conserved among all homologs, then it is very likely that this specific run is used for purposeful transcriptional slippage to generate a set of heterogeneous mRNAs. Subsequent translation of such mRNAs will result in the synthesis of more than one protein product from the same gene. An example is shown in Figure 6 for IS elements from *D. radiodurans*. We have not found homologous IS elements that contain insertions or deletions in the run of As. Those shown on Figure 6 are the only homologs found.

In general, a conserved run of As or Ts in several homologs does not imply that replication slippage is impossible on such a run. For example, when insertion of an additional nucleotide is deleterious, there will be selection against sequences with the additional nucleotide. However, in this case such replicational slippage cannot be referred to as being functional.

The comparative sequence analysis of genes with runs of nine As and Ts from genomes where such repeat bases are slippage-prone, revealed *S. aureus mapW *as a candidate for functional utilization of transcriptional slippage. *mapW *belongs to a group of *map *genes encoding MHC class II (major histocompatibility complex class II)-like proteins. *mapW *consists of two ORFs and it was proposed earlier that they can be expressed together to produce a full-length 'fusion' protein [38]. Perhaps the ability of *S. aureus *to encode MHC-II like proteins with variable length can facilitate survival in mammals of varied genetic backgrounds [39]. However, the presence of *mapW *genes with an uninterrupted ORF in some *S. aureus *strains suggests that replicational slippage can be also utilized in this case.

The largest group of functionally utilized transcriptional slippage sites belongs to mobile IS elements. We have found patterns of 9 As in 27 IS elements from the following organisms - *Deinococcus radiodurans*, *Mesorhizobium loti*, *Nostoc *sp. PCC 7120, *Streptococcus pyogenes *and *Sulfolobus solfataricus*. Interestingly, some homologous IS elements from *D. radiodurans *and *Nostoc *sp. PCC 7120 have 8As instead of 9As in the same location. This suggests that in these organisms, transcriptional slippage is productive even on eight As. Figure 6 illustrates codon alignment of homologous IS elements from *D. radiodurans*. It is clear that the stretch of As is evolutionally preserved among these IS elements (although its length varies, there is no deletion or insertions) and their ORF organization suggests that runs of As are used to produce ORF fusions. (A high-resolution FITC mass spectrometric analysis of numerous tryptic peptides from *D. radiodurans *has been performed by Smith and colleagues [40]. A preliminary analysis of these data is revealing products of IS element mRNAs synthesized via transcriptional slippage (R. Smith, P.V.B., A.W.H, J.Z, R.F.G. and J.F.A, unpublished results) Alignment of IS elements from *Nostoc *is not shown, as all its elements are identical except for the length of the poly(A) run varying from 8 to 10 As. Many IS elements encode their transposase in two overlapping ORFs, *orfA *and *orfB*. Synthesis of a fused ORFA-ORFB product is required for transposition. The most common known mechanism for synthesis of ORFA-ORFB fusion is -1 ribosomal frameshifting (see [41-44] for reviews). Transcriptional slippage has, however, been proposed previously as an alternative mechanism for one IS element [19]. The present study has identified a number of IS elements utilizing transcriptional slippage for synthesis of their ORFA-ORFB fusion. Therefore transcriptional slippage can be considered as a common mechanism for IS element expression.

In addition, we have found a set of pseudo pseudogenes where what is normally considered as a frameshift mutation extends a non-slippery pattern of 8 As to the slippage-prone sequence of 9 As. As a result, such a frameshift mutation does not lead to full inactivation of a gene that normally could be annotated as a pseudogene, as a normal functional product is still produced. The advantage of the unusual decoding of these genes by transcriptional slippage, compared to standard decoding of wild-type counterparts, is uncertain. It is clear that such cases were generated by single mutations and they may, or may not, be present in different isolates from the same species. Transcriptional slippage can, however, be considered as functionally utilized, since if such genes were transcribed, a proportion of the mRNA synthesized should contain the intact coding information. This important consideration needs to be taken in account in genome annotation.

Although organism-specific utilization of transcriptional slippage cannot be ruled out, we have identified a large number of genes where, using comparative analysis, no apparent functional role can be assigned for transcriptional slippage. This result is parallel to our previous analysis of frameshift-inducing sequences in the *E. coli *K12 genome [31]. It was shown that a significant level of frameshifting errors occur in many *E. coli *genes containing A_AAA_AAG sequences (codons are separated by underscoring), but no such sequences were found in highly expressed genes [31]. Similar considerations can be applied here for transcriptional slippage. When erroneous nonstandard decoding occurs in genes that are not highly expressed, the cellular load is modest owing to the low level of aberrant product compared to the total protein mass. Such situations may be easily tolerated.

Transcriptional slippage motifs were found in many ORFans, but any functional purpose could not be assessed in the present study. We found runs of 9A or 9T in 48 ORFans. The origin(s) of ORFans is mysterious. While some of them are likely to be 'coincidental ORFs' or 'junk ORFans' which do not produce proteins under any conditions [45-47], many ORFans are likely to be real genes [36,48,49].

**Figure 7:**
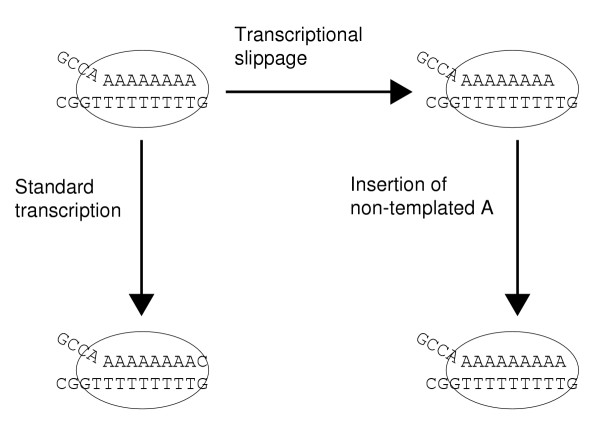
A model of transcriptional slippage.

The analysis of transcriptional slippage in this study was limited to that occurring on 9As and 9Ts. It is clear, however, that the efficiency of transcriptional slippage on runs of As and Ts is highly organism dependent, and there are a number of bacteria in which transcriptional slippage may occur on runs of shorter length. In addition, transcriptional slippage patterns can occur on other nucleotide repeats. The simplest mechanism that can be proposed for transcriptional slippage is dissociation of the growing RNA chain from its DNA template while inside an open RNA polymerase complex, and subsequent re-association with the DNA template at a new location (Figure 7). On this basis, other repeat patterns of low complexity are likely to result in transcriptional slippage. For example, (AT)*n *may result in insertion of additional non-templated ATs. Transcriptional slippage sites can be also formed by combination of two relatively short homopolymeric patterns as in paramyxoviruses [14].

Simple sequence repeats (SSR), also known as microsatellites, occur frequently in virulence genes of different pathogenic bacteria [37,50,51]. Because of replicational slippage, they are responsible for hypermutability and phase variations in pathogenic bacteria [52]. The effect of such sequences on transcription and translation has not yet been extensively studied. Such sequences could also result in nonstandard decoding (transcriptional slippage or ribosomal frameshifting) and consequently express more than one protein product. Expression of multiple products encoded by virulence genes may be beneficial for pathogens as a strategy for evading the host immune response. Statistical, experimental and functional analysis of such sequences in relation to transcription and translation will hopefully be the subject of further investigation.

## Materials and methods

### Analysis of A and T repeat distribution in bacterial genomes

Fasta files containing nucleotide sequences of entire bacterial genomes and nucleotide sequences of coding regions were downloaded from the National Center for Biotechnology Information ftp site [53] on 25 March, 2003. Occurrences of A and T runs with different lengths were calculated for each genome in the file containing genomic sequences (accession_number.fna) and in the files containing nucleotide sequences of coding ORFs (accession_number.ffn). The ratios of occurrences of runs of A and T between .fna files and .ffn files were calculated for every accession number and the data are summarized in 1.

Random genomes were generated for representative genomes as described in [31]. In addition we applied DiShuffle and CodonDiShuffle programs provided by C. Burge [32]. The ratios between occurrences of A and T runs in real genomes and the mean values for A and T runs in random genomes were further calculated.

### Generation of novel protein sequences corresponding to those produced via transcriptional slippage

Runs of 9A or 9Ts were sought within coding regions of genomic sequences of completed bacterial genomes. To generate a novel *in silico *protein that can be produced by transcriptional slippage, one and two As or Ts were introduced into the pattern of 9As or 9Ts. The length of the resulting ORF in these sequences was compared to the ORF in the original sequences. Those sequences that contain ORFs longer than the original were selected for further analysis.

## Additional data files

Additional data is available with the online version of this paper. Additional data file 1 contains numbers of occurrences of A and T runs in bacterial genomes. Additional data file 2 contains information about genes where 9A or 9T patterns were found.

## Supplementary Material

Additional File 1 Numbers of occurrences of A and T runs in bacterial genomes. Column A is used for the names of analyzed files and row 1 indicates the length of A/T run. Sheet 'whole genomes' corresponds to occurrences in entire genomes, sheet 'coding sequences' corresponds to occurrences in coding regions and sheet 'ATRatio' corresponds to the ratio between these numbers Click here for file

Additional File 2 Information about genes where 9A or 9T patterns were found. These genes correspond to the pool of genes selected for comparative analysis. The table contains representatives from 98 gene groups selected in step 4 of the scheme in Figure 3. Column A is used for accession numbers. B is for coordinates of the corresponding gene. C indicates whether it is a run of A or T in a sense strand. D shows the functional status of a gene. The functional status is annotated by text and by color. The red color is used for genes with potential positive role of transcriptional slippage, blue is for those where there is no positive functional role, green is for genes where transcriptional slippage might restore a disrupted ORF and grey is used for ORFans, where functional status cannot be assessed. Column E contains nucleotide sequences of mRNAs produced *via *transcriptional slippage with ORFs longer than those in the original DNA templates. Column F contains the corresponding protein sequences Click here for file

## References

[B1] Chamberlin M, Berg P (1962). Deoxyribonucleic acid-directed synthesis of ribonucleic acid by an enzyme from *Escherichia coli*.. Proc Natl Acad Sci USA.

[B2] Wagner LA, Weiss RB, Driscoll R, Dunn DS, Gesteland RF (1990). Transcriptional slippage occurs during elongation at runs of adenine or thymine in *Escherichia coli*.. Nucleic Acids Res.

[B3] Liu C, Heath LS, Turnbough CL (1994). Regulation of *pyrBI *operon expression in *Escherichia coli *by UTP-sensitive reiterative RNA synthesis during transcriptional initiation.. Genes Dev.

[B4] Qi F, Turnbough CL (1995). Regulation of *codBA *operon expression in *Escherichia coli *by UTP-dependent reiterative transcription and UTP-sensitive transcriptional start site switching.. J Mol Biol.

[B5] van Leeuwen FW, de Kleijn DP, van den Hurk HH, Neubauer A, Sonnemans MA, Sluijs JA, Koycu S, Ramdjielal RD, Salehi A, Martens GJ (1998). Frameshift mutants of beta amyloid precursor protein and ubiquitin-B in Alzheimer's and Down patients.. Science.

[B6] Martin GM, Bressler SL (2000). Transcriptional infidelity in aging cells and its relevance for the Orgel hypothesis.. Neurobiol Aging.

[B7] van Leeuwen FW, Fischer DF, Kamel D, Sluijs JA, Sonnemans MA, Benne R, Swaab DF, Salehi A, Hol EM (2000). Molecular misreading: a new type of transcript mutation expressed during aging.. Neurobiol Aging.

[B8] van Leeuwen FW (2004). Neuropeptide research discloses part of the secrets of Alzheimer's disease neuropathogenesis: state of the art 2004.. Neurosci Lett.

[B9] Laken SJ, Petersen GM, Gruber SB, Oddoux C, Ostrer H, Giardiello FM, Hamilton SR, Hampel H, Markowitz A, Klimstra D (1997). Familial colorectal cancer in Ashkenazim due to a hypermutable tract in APC.. Nat Genet.

[B10] Raabe M, Linton MF, Young SG (1998). Long runs of adenines and human mutations.. Am J Med Genet.

[B11] Linton MF, Pierotti V, Young SG (1992). Reading-frame restoration with an apolipoprotein B gene frameshift mutation.. Proc Natl Acad Sci USA.

[B12] Linton MF, Raabe M, Pierotti V, Young SG (1997). Reading-frame restoration by transcriptional slippage at long stretches of adenine residues in mammalian cells.. J Biol Chem.

[B13] Benson KF, Person RE, Li FQ, Williams K, Horwitz M (2004). Paradoxical homozygous expression from heterozygotes and heterozygous expression from homozygotes as a consequence of transcriptional infidelity through a polyadenine tract in the AP3B1 gene responsible for canine cyclic neutropenia.. Nucleic Acids Res.

[B14] Hausmann S, Garcin D, Delenda C, Kolakofsky D (1999). The versatility of paramyxovirus RNA polymerase stuttering.. J Virol.

[B15] Iseni F, Baudin F, Garcin D, Marq JB, Ruigrok RW, Kolakofsky D (2002). Chemical modification of nucleotide bases and mRNA editing depend on hexamer or nucleoprotein phase in Sendai virus nucleocapsids.. RNA.

[B16] Volchkov VE, Becker S, Volchkova VA, Ternovoj VA, Kotov AN, Netesov SV, Klenk HD (1995). GP mRNA of Ebola virus is edited by the Ebola virus polymerase and by T7 and vaccinia virus polymerases.. Virology.

[B17] Sanchez A, Trappier SG, Mahy BW, Peters CJ, Nichol ST (1996). The virion glycoproteins of Ebola viruses are encoded in two reading frames and are expressed through transcriptional editing.. Proc Natl Acad Sci USA.

[B18] Volchkov VE, Volchkova VA, Muhlberger E, Kolesnikova LV, Weik M, Dolnik O, Klenk HD (2001). Recovery of infectious Ebola virus from complementary DNA: RNA editing of the GP gene and viral cytotoxicity.. Science.

[B19] Larsen B, Wills NM, Nelson C, Atkins JF, Gesteland RF (2000). Nonlinearity in genetic decoding: homologous DNA replicase genes use alternatives of transcriptional slippage or translational frameshifting.. Proc Natl Acad Sci USA.

[B20] Yurieva O, Skangalis M, Kuriyan J, O'Donnell M (1997). *Thermus thermophilis dnaX *homolog encoding gamma- and tau-like proteins of the chromosomal replicase.. J Biol Chem.

[B21] Bullard JM, Williams JC, Acker WK, Jacobi C, Janjic N, McHenry CS (2002). DNA polymerase III holoenzyme from *Thermus thermophilus *identification, expression, purification of components, and use to reconstitute a processive replicase.. J Biol Chem.

[B22] Flower AM, McHenry CS (1990). The gamma subunit of DNA polymerase III holoenzyme of *Escherichia coli *is produced by ribosomal frameshifting.. Proc Natl Acad Sci USA.

[B23] Tsuchihashi Z, Kornberg A (1990). Translational frameshifting generates the gamma subunit of DNA polymerase III holoenzyme.. Proc Natl Acad Sci USA.

[B24] Blinkowa AL, Walker JR (1990). Programmed ribosomal frameshifting generates the *Escherichia coli *DNA polymerase III gamma subunit from within the tau subunit reading frame.. Nucleic Acids Res.

[B25] Blinkowa AL, Walker JR (1990). Programmed ribosomal frameshifting generates the *Escherichia coli *DNA polymerase III gamma subunit from within the tau subunit reading frame.. Nucleic Acids Res.

[B26] Baranov PV, Gesteland RF, Atkins JF (2002). Recoding: translational bifurcations in gene expression.. Gene.

[B27] Penno C, Sansonetti P, Parsot C (2005). Frameshifting by transcriptional slippage is involved in production of MxiE, the transcription activator regulated by the activity of the type III secretion apparatus in *Shigella flexneri*.. Mol Microbiol.

[B28] Wheeler DL, Church DM, Edgar R, Federhen S, Helmberg W, Madden TL, Pontius JU, Schuler GD, Schriml LM, Sequeira E (2004). Database resources of the National Center for Biotechnology Information: update.. Nucleic Acids Res.

[B29] Field D, Wills C (1998). Abundant microsatellite polymorphism in *Saccharomyces cerevisiae*, and the different distributions of microsatellites in eight prokaryotes and *S. cerevisiae*, result from strong mutation pressures and a variety of selective forces.. Proc Natl Acad Sci U S A.

[B30] Wilson KS, von Hippel PH (1995). Transcription termination at intrinsic terminators: the role of the RNA hairpin.. Proc Natl Acad Sci USA.

[B31] Gurvich OL, Baranov PV, Zhou J, Hammer AW, Gesteland RF, Atkins JF (2003). Sequences that direct significant levels of frameshifting are frequent in coding regions of *Escherichia coli*.. EMBO J.

[B32] Katz L, Burge CB (2003). Widespread selection for local RNA secondary structure in coding regions of bacterial genes.. Genome Res.

[B33] Altschul SF, Gish W, Miller W, Myers EW, Lipman DJ (1990). Basic local alignment search tool.. J Mol Biol.

[B34] Fischer D, Eisenberg D (1999). Finding families for genomic ORFans.. Bioinformatics.

[B35] Siew N, Azaria Y, Fischer D (2004). The ORFanage: an ORFan database.. Nucleic Acids Res.

[B36] Metzgar D, Bytof J, Wills C (2000). Selection against frameshift mutations limits microsatellite expansion in coding DNA.. Genome Res.

[B37] Parkhill J, Wren BW, Mungall K, Ketley JM, Churcher C, Basham D, Chillingworth T, Davies RM, Feltwell T, Holroyd S (2000). The genome sequence of the food-borne pathogen *Campylobacter jejuni *reveals hypervariable sequences.. Nature.

[B38] Kuroda M, Ohta T, Uchiyama I, Baba T, Yuzawa H, Kobayashi I, Cui L, Oguchi A, Aoki K, Nagai Y (2001). Whole genome sequencing of meticillin-resistant *Staphylococcus aureus*.. Lancet.

[B39] Lee LY, Miyamoto YJ, McIntyre BW, Hook M, McCrea KW, McDevitt D, Brown EL (2002). The *Staphylococcus aureus *Map protein is an immunomodulator that interferes with T cell-mediated responses.. J Clin Invest.

[B40] Lipton MS, Pasa-Tolic L, Anderson GA, Anderson DJ, Auberry DL, Battista JR, Daly MJ, Fredrickson J, Hixson KK, Kostandarithes H (2002). Global analysis of the *Deinococcus radiodurans *proteome by using accurate mass tags.. Proc Natl Acad Sci USA.

[B41] Chandler M, Fayet O (1993). Translational frameshifting in the control of transposition in bacteria.. Mol Microbiol.

[B42] Ohtsubo F, Sekine Y (1996). Bacterial insertion sequences.. Curr Top Microbiol Immunol.

[B43] Farabaugh PJ (1997). Programmed Alternative Reading of the Genetic Code.

[B44] Mahillon J, Chandler M (1998). Insertion sequences.. Microbiol Mol Biol Rev.

[B45] Dujon B, Alexandraki D, Andre B, Ansorge W, Baladron V, Ballesta JP, Banrevi A, Bolle PA, Bolotin-Fukuhara M, Bossier P (1994). Complete DNA sequence of yeast chromosome XI.. Nature.

[B46] Goffeau A, Barrell BG, Bussey H, Davis RW, Dujon B, Feldmann H, Galibert F, Hoheisel JD, Jacq C, Johnston M (1996). Life with 6000 genes.. Science.

[B47] Monchois V, Abergel C, Sturgis J, Jeudy S, Claverie JM (2001). *Escherichia coli ykfE *ORFan gene encodes a potent inhibitor of C-type lysozyme.. J Biol Chem.

[B48] Daubin V, Ochman H (2004). Bacterial genomes as new gene homes: the genealogy of ORFans in *E. coli*.. Genome Res.

[B49] Siew N, Fischer D (2003). Twenty thousand ORFan microbial protein families for the biologist?. Structure.

[B50] Peak IR, Jennings MP, Hood DW, Bisercic M, Moxon ER (1996). Tetrameric repeat units associated with virulence factor phase variation in *Haemophilus *also occur in *Neisseria *spp. and *Moraxella catarrhalis*.. FEMS Microbiol Lett.

[B51] Karlin S, Mrazek J, Campbell AM (1997). Compositional biases of bacterial genomes and evolutionary implications.. J Bacteriol.

[B52] Roche RJ, Moxon ER (1995). Phenotypic variation *in Haemophilus influenzae*: the interrelationship of colony opacity, capsule and lipopolysaccharide.. Microb Pathog.

[B53] NCBI ftp site. ftp://ftp.ncbi.nlm.nih.gov/genomes/Bacteria/.

[B54] Thompson JD, Gibson TJ, Plewniak F, Jeanmougin F, Higgins DG (1997). The CLUSTAL_X windows interface: flexible strategies for multiple sequence alignment aided by quality analysis tools.. Nucleic Acids Res.

